# Non-invasive prenatal diagnosis using massively parallel sequencing - first experience in Germany

**DOI:** 10.1186/1755-8166-7-S1-I14

**Published:** 2014-01-21

**Authors:** Rolf-Dieter Wegner, Markus Stumm, Wera Hofmann

**Affiliations:** 1 Zentrum für Pränataldiagniostik & Humangenetik – Kudamm 199, Berlin, Germany; 2 BG Berlin Genetics GmbH, Berlin, Germany; 3 LifeCodexx AG, Konstanz, Germany

## 

Non-invasive prenatal diagnosis (NIPD) of aneuploidies by cell free fetal DNA (cff-DNA) from maternal plasma has reached the reliability to be applied in a clinical setting. It started with an observation that fetal DNA fragments are present in the blood of pregnant women. One main obstacle to be resolved had been the low concentration of cff-DNA among maternal DNA fragments, in general 2 – 10 % at 10^th^ week of pregnancy. This problem was resolved by the technical development of next generation sequencing applying massively parallel sequencing of millions of DNA fragments out of a single blood sample of 10 ml followed by bioinformatic processing of the data. Algorithms for calculations of z-scores were validated to allow a highly accurate distinction of pregnancies with an euploid fetus from those with a fetus carrying certain aneuploidies. With focus on the reliability of results it should be in mind that the cff-DNA is derived from the cytotrophoblast, e.g. from the placental tissue which is analyzed in CVS short term culture. Hitherto collected clinical data show sensitivity and specificity in agreement with a diagnostic test.

In Germany, aneuploidy testing by NIPD started in 2012. Initially, the approach allowed the detection of trisomy 21. However, meanwhile probing for trisomy 13, trisomy 18 and the sex chromosome constitution is feasible. At the moment, the test is indicated for women with singleton pregnancies at an increased risk for aneuploidies. Blood samples should not be taken before 9+0 week of pregnancy. However, it is suggested to perform NIPD only in conjunction with a first trimester ultrasonographic examination for a profound judging of the fetal situation. For the time being, testing time is reduced to 10 working days or even less.

In Germany, by now more than 4000 tests had been performed. In Berlin, data exceeding 250 cases had been collected by BG Berlin Genetics. The data of NIPD will be discussed in comparison to invasive prenatal diagnosis.

